# Construction and application of fetal loss risk model in systemic lupus erythematosus patients with mild disease severity

**DOI:** 10.1186/s12884-024-06679-6

**Published:** 2024-07-23

**Authors:** Yanran Chen, Yanjuan Chen, Bo Li, Wengyi Xu, Peipei Lei, Hongyang Liu, Dongzhou Liu, Xiaoping Hong

**Affiliations:** 1 The Second Clinical Medical College, Jinan University (Shenzhen People’s Hospital), Shenzhen, China; 2 Department of Rheumatology and Immunology, Shenzhen People’s Hospital, The Second Clinical Medical College, Jinan University, The First Affiliated Hospital, Southern University of Science and Technology, Shenzhen, China; 3 Department of Rheumatology and Immunology, Shenzhen Longhua people’s Hospital, Shenzhen, China

**Keywords:** Systemic lupus erythematosus, Mild disease severity, Pregnancy outcome, Fetal loss, Prediction nomogram

## Abstract

**Background:**

This dynamic nomogram model was developed to predict the probability of fetal loss in pregnant patients with systemic lupus erythematosus (SLE) with mild disease severity before conception.

**Methods:**

An analysis was conducted on 314 pregnancy records of patients with SLE who were hospitalized between January 2015 and January 2022 at Shenzhen People's Hospital, and the Longhua Branch of Shenzhen People's Hospital. Data from the Longhua Branch of the Shenzhen People's Hospital were utilized as an independent external validation cohort. The nomogram, a widely used statistical visualization tool to predict disease onset, progression, prognosis, and survival, was created after feature selection using multivariate logistic regression analysis. To evaluate the model prediction performance, we employed the receiver operating characteristic curve, calibration curve, and decision curve analysis.

**Results:**

Lupus nephritis, complement 3, immunoglobulin G, serum albumin, C-reactive protein, and hydroxychloroquine were all included in the nomogram model. The model demonstrated good calibration and discriminatory power, with an area under the curve of 0.867 (95% confidence interval: 0.787–0.947). According to decision curve analysis, the nomogram model exhibited clinical importance when the probability of fetal loss in patients with SLE ranged between 10 and 70%. The predictive ability of the model was demonstrated through external validation.

**Conclusion:**

The predictive nomogram approach may facilitate precise management of pregnant patients with SLE with mild disease severity before conception.

**Supplementary Information:**

The online version contains supplementary material available at 10.1186/s12884-024-06679-6.

## Background

Systemic lupus erythematosus (SLE) is a chronic, relapsing, remitting autoimmune disease that primarily affects women during their reproductive years [1, 2]. Fluctuations in disease activity are a clinical feature of SLE, with periods of high disease activity followed by those of low activity [3]. For patients of childbearing age with fertility requirements, whether SLE increases the risk of adverse pregnancy outcomes (APO) is a major concern [4]. The relationship between SLE and APO has been a significant topic of discussion, particularly regarding the impact of SLE on fetal loss [5]. The pregnancy outcomes in the SLE group included high rates of fetal loss, with an estimated 20% of pregnancies terminating in miscarriages and 3% in stillbirths [6, 7]. Preconception counseling and management of pregnancies in patients with SLE are recommended, particularly to evaluate disease activity and determine the optimal timing of conception [8]. Despite conflicting findings from numerous studies, risk factors such as antiphospholipid syndrome (APS) and high active lupus activity scores 6 months before conception have been linked to fetal loss in women with SLE [9–13]. Accordingly, clinicians providing preconception counseling will pay extra attention to patients with SLE presenting with the risk factors. However, few studies have focused on pregnancy outcomes in pregnant women with SLE who have mild disease severity before conception and are not diagnosed with APS [14]. The risk of fetal loss in patients with SLE, who are not classified as having severe disease may be underestimated. In addition to providing effective prenatal counseling and treatment to patients with SLE, predicting pregnancy outcomes could also improve sensitivity to fetal loss [15]. Most of the studies were based on a single-center cohort construction. The factors influencing the studies included a low pre-inclusion of baseline data, unclear strength of predictor effects on outcomes, low clinical tractability, and clinical fit of predictors yet to be validated [16]. Due to the extent of research and variable findings, few explicit recommendations are available for SLE fertility risk assessment in the current treatment guidelines. In contemporary clinical practice, the assessment of pregnancy in patients is mainly empirical.

The European League Against Rheumatism (EULAR) published recommendations on the management of SLE in 2019, stratifying the disease severity into mild, moderate, and severe [17]. To date, no risk assessment model is available for evaluating the risk of fetal loss in pregnant women with SLE and mild disease severity. Nomograms are increasingly used in predictive modeling due to their simplicity, intuitiveness, and advanced capabilities. A dynamic nomogram is a simple web-based graphical tool that incorporates several important factors and is useful for personalized risk assessment. In this study, we developed a clinical prediction model for fetal loss in patients with SLE with mild disease severity based on a retrospective analysis of patient data from a hospital in China. Moreover, we externally validated the model using an independent cohort.

## Methods

### Study design and population

This observational, retrospective, two-center cohort study was approved by the Ethics Committee of Shenzhen People's Hospital, China (LL-KT 2019066). Additional data were obtained through electronic medical record reviews and personal interviews when necessary. Owing to the retrospective nature of this study, the requirement for informed consent was waived, and verbal informed consent was obtained from the patients for personal interviews. The study population was selected from 299 pregnant women with SLE who were followed up at Shenzhen People's Hospital and the Longhua Branch of Shenzhen People's Hospital between 2015 and 2022. The medical records of 314 potentially eligible pregnancies from 299 patients with SLE were screened and logged, with each pregnancy considered and evaluated individually.

Individuals that met the inclusion criteria of 1) age ≥ 18 years, 2) diagnosed with SLE according to the 1997 American College of Rheumatology (ACR) [17], the 2012 Systemic Lupus International Collaboration Clinic Classification criteria [18] or the EULAR/ACR-2019 criteria [19], 3) received at least one visit at a rheumatology department in the 6 months before conception and regular visits during pregnancy, 4) had mild disease severity of lupus before conception, with disease activity assessed based on the 2019 update of the EULAR recommendations for the management of SLE [16]. Mild disease severity was defined as constitutional symptoms including mild arthritis, rash ≤ 9%, platelets (PLTs) count 50–100 × 103/mm3, SLE disease activity index (SLEDAI) ≤ 6 [20]; British Isles Lupus Assessment Group (BILAG) C or BILAG B manifestation [20]. The study population was limited to patients with low disease severity before conception; thus, patients with new-onset lupus during pregnancy were excluded. We also excluded patients with multiple pregnancies and APS due to their association with more APOs, aiming to eliminate the potential confounding effects. Elective termination of pregnancy due to personal reasons is also excluded.

**Fig. 1:**
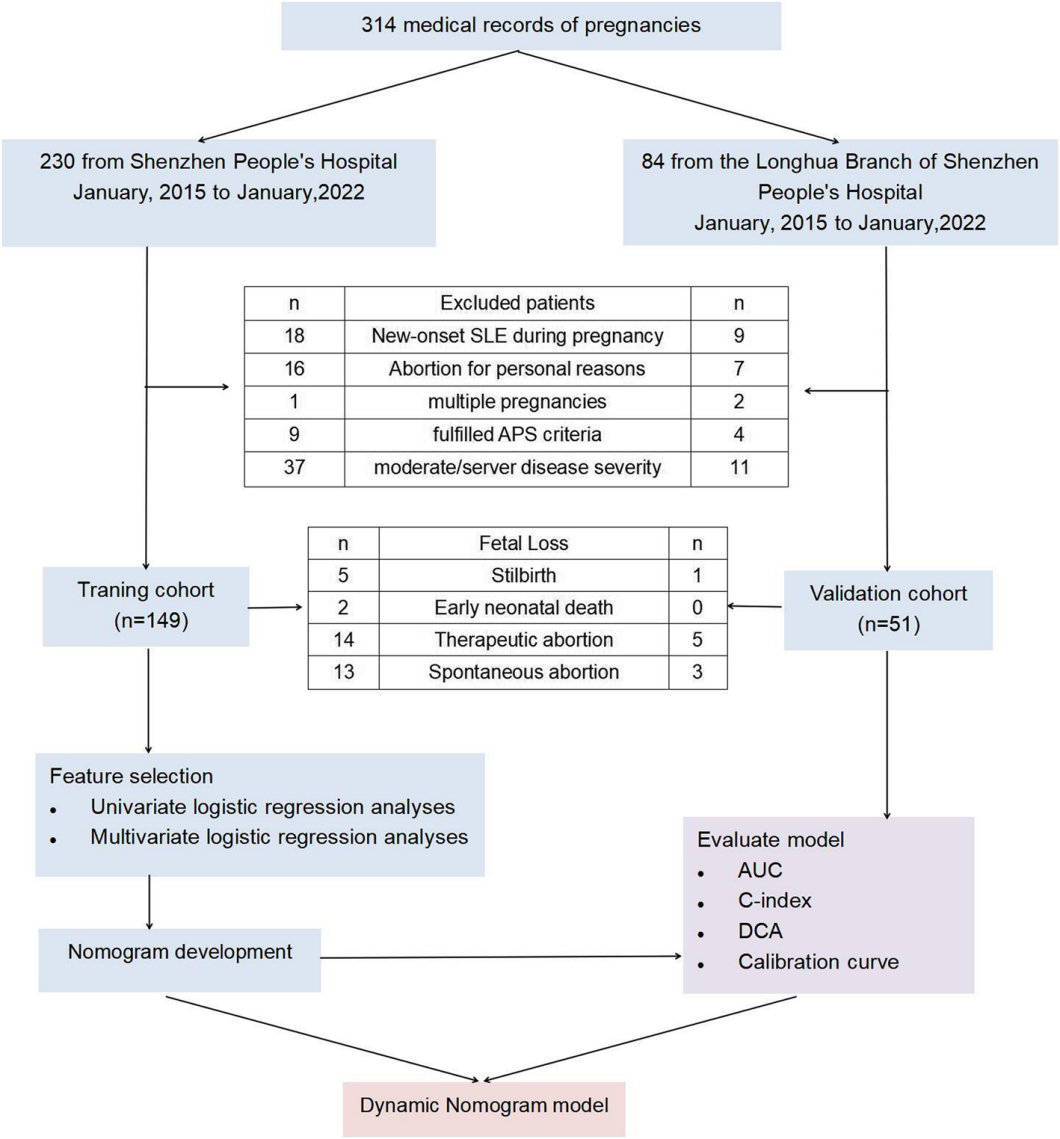
Flow chart for patient selection

After screening and evaluation, the records of 200 pregnancies from 189 patients with SLE who met the inclusion criteria and did not fulfill any of the exclusion criteria were included in the study. The training cohort included 149 pregnancy records from 138 patients with SLE who were hospitalized between January 1, 2015, and January 1, 2022, at Shenzhen People's Hospital. In the external validation cohort, 51 pregnancy records of patients with SLE were collected at the Longhua Branch of Shenzhen People's Hospital from January 1, 2015, to January 1, 2022. The training cohort included an analysis of 149 pregnancy outcomes among 138 patients with SLE. Among them were 34 cases of fetal loss, with the remaining 115 cases resulting in live births, equating to a fetal loss rate of 22.8%. The validation cohort analyzed 51 pregnancy outcomes in 51 patients with SLE. Among them, nine cases were of fetal loss, while the remaining 42 cases resulted in live births, resulting in a fetal loss rate of 17.6%. The flowchart is displayed in Fig. 1.

### Data collection

Follow-up records of the women throughout pregnancy were retrieved using electronic medical records to document laboratory markers measured for the first time during pregnancy and to assess their correlation with the pregnancy outcomes. Clinical baseline data included age, domicile, body mass index before conception, and medical history. SLE-related data included age at the time of diagnosis, disease duration, and previous systemic involvement (arthritis, cutaneous lesions, hematological disorders, serositis, and lupus nephritis). Pregnancy history included whether the pregnancy was primiparous, whether a history of SLE-related therapeutic miscarriage was present, as well as a history of two or more recurrent miscarriages. The following laboratory data were collected: levels of hemoglobin, white blood cells, PLTs, C-reactive protein (CRP), urine protein (PRO), 24-h PRO (24-h PRO), hematocrit (ESR), albumin (ALB), cholesterol, triglycerides, low-density lipoprotein (LDL), high-density lipoprotein (HDL), immunoglobulin (Ig) A, IgM, IgG, complement 3 (C3), and complement 4 (C4). The immunological data included antinuclear antibodies, anti-dsDNA antibodies, anti-Smith antibodies (anti-Sm), anti-ro/SSA antibodies, anti-la/SSB antibodies, anticardiolipin (aCL) antibodies against IgM and IgG, and lupus anticoagulants. We gathered information on the patients’ pregnancy medication history, which included glucocorticoids, hydroxychloroquine (HCQ), immunosuppressants, and aspirin. Immunosuppressants included cyclosporine A, tacrolimus, mofetil, azathioprine, and methotrexate. All laboratory tests were performed using standardized methods. We collected 44 clinical and laboratory indicators from 200 patients in the study as potential predictors of fetal loss in pregnant women with SLE. The data for the training and validating cohorts are presented in Table 1. Due to the small amount of missing data for the primary outcome (i.e., less than 1 per cent of the dataset), multiple imputation was not implemented.

**Table 1:** Baseline characteristics of training cohort and validation cohort

Factor	Category	Training Cohort (*n* = 149)	Validation Cohort (*n* = 51)	*P* value
Fetal outcomes	Live birth	115(77.2)	42(82.3)	0.858
n(%)	Fetal loss	34(22.8)	9(17.6)	
Baseline characteristics	Age(year),mean (S.D.)	29.8(4.81)	29.9(3.31)	0.656
	BMI before pregnant (kg/m2),mean (S.D.)	21.58(2.67)	22.06(3.16)	0.311
Other chronic disease	Prepregnancy diabetes	5(3.4)	2(3.9)	0.344
	Prepregnancy hypertension	7(4.7)	3(5.9)	0.661
Previous SLE clinical features	Age of SLE diagnosis(years)	23(21–27)	25(20–28)	0.123
median (IQR)	SLE duration at conception (years)	4(2–6)	4(2–4)	0.502
System manifestation	Arthritis	26(17.4)	11(21.5)	0.109
n(%)	Cutaneous lesion	63(42.3)	29(56.8)	0.009
	Hematological disorder	23(15.4)	8(15.6)	0.233
	Serositis	6(4.0)	4(7.8)	0.078
	Nephritis	35(23.5)	11(21.6)	0.306
	SLADAI before conception, median (IQR)	4(2–6)	2(2–4)	0.062
Obstetric history	Primigravida	43(28.9)	18(35.3)	0.122
n(%)	History of therapeutic abortion	22(14.8)	13(17.65)	0.238
	History of recurrent miscarriages ≥ 2	19(12.8)	9(17.6)	0.511
Laboratory data	ANA positive	135(90.6)	42(82.3)	0.138
n(%)	Anti-Ro/SSA positive	68(45.6)	23(45.1)	0.488
	Anti-La/ SSB positive	21(14.1)	15(29.4)	0.011
	Anti-dsDNA positive	60(40.3)	20(39.2)	0.873
	Anti-Sm positive	36(24.2)	7(13.7)	0.143
	C3 (g/L),mean (S.D.)	0.91(0.26)	0.83 (0.31)	0.126
	C4 (g/L),mean (S.D.)	0.2(0.49)	0.16 (0.10)	0.625
	Lupus anticoagulant positive	37(24.8)	11(21.6)	0.759
	aCL-IgG positive	17(11.4)	9(17.6)	0.934
	aCL-IgM positive	8(5.4)	3(5.8)	0.233
Hyperimmunoglobulin	IgA (g/L)	2.44(1.09)	2.24 (0.84)	0.295
mean (S.D.)	IgG (g/L)	12.79(4.27)	13.63 (4.81)	0.239
	IgM(g/L)	1.00(0.54)	0.96(0.45)	0.266
	HB (g/L)	108.19(17.21)	112.88 (12.81)	0.870
	WBC(× 109/L)	7.31(3.21)	7.99 (2.97)	0.890
	PLT(× 109/L)	180.92(81.84)	198.08 (76.53)	0.427
	Serum albumin(g/L)	33.65(4.85)	37.45 (5.77)	0.025
	ESR (mm/h)	33.46(26.51)	32.08 (23.86)	0.808
	CRP(mg/L)	11.68(8.84)	15.99 (9.72)	0.821
	24-h PRO(g/L)	0.8(1.44)	0.98 (1.96)	0.447
	PRO,n(%)	57(38.3)	13(25.6)	0.131
	TC (mmol/L)	4.28(1.65)	3.82(2.13)	0.008
	TG (mmol/L)	2.05(1.24)	2.34(1.62)	0.090
	HDL-C(mmol/L)	1.38(0.39)	1.45 (0.44)	0.072
	LDL-C(mmol/L)	2.25(1.18)	2.32 (1.04)	0.117
Drugs taken at the onset of pregnancy	Prednisone	66(44.3)	27(52.9)	0.787
n(%)	Hydroxychloroquine	71(47.7)	30(58.8)	0.102
	Immunosuppressants	34(22.8)	4(7.8)	0.250
	Aspirin	13(8.7)	2(3.9)	0.298

*IQR* interquartile range, *BMI* body mass index, *SLEDAI-2 k* Systemic Lupus Erythematosus Disease Activity Index -2 K, *ANA* antinuclear antibodies, *C3* hypocomplementania C3, *C4* hypocomplementania C4, *aCL-IgG* anticardiolipin- immunoglobulin G, *aCL-IgM* anticardiolipin-immunoglobulin M, *IgA* immunoglobulin A, *IgG* immunoglobulin G, *IgM* immunoglobulin M, *HB* hemoglobin, *WBC* white blood cell, *PLT* platelet, *ESR* erythrocyte sedimentation rate, *CRP* C-reactive protein, *PRO* urine protein, *24-h PRO* 24-h urine protein, *TC* totalcholesterol, *TG* triglyceride, *HDL* high-density lipoprotein, *LDL* low-density lipoprotein

### Definition of fetal and obstetric outcomes

Live birth was defined as the delivery of a live infant at ≥ 20 weeks of gestation, with the newborn surviving for more than 6 days. The primary fetal loss outcome was defined as all pregnancies without a live birth, including spontaneous abortion (spontaneous termination of pregnancy before 20 weeks of gestation), therapeutic abortion (elective termination of pregnancy resulting from the presence of potentially life-threatening maternal health, such as life-threatening lupus flare), stillbirth (intrauterine spontaneous fetal demise after 20 weeks of gestation), or early neonatal death (neonatal death within 7 days of delivery). Additionally, APOs included fetal and maternal outcomes. Adverse fetal outcomes included fetal death, neonatal death, fetal growth restriction, admission to the neonatal intensive care unit, preterm birth (< 37 weeks), and fetal growth restriction. Adverse maternal outcomes included lupus flares, emergency cesarean sections, and preeclampsia.

### Feature selection and nomogram model establishment

Univariate and multivariate logistic regression analyses were used to analyze the risk factors associated with fetal loss in pregnant women with SLE. When the univariate logistic analysis was statistically significant (*P* < 0.05), these factors were incorporated into multivariate logistic regression analysis. We used multivariate logistic regression analysis to determine the independent factors associated with fetal loss. Based on the results of the logistic regression, we identified the independent correlation factors and constructed a nomogram prediction model. In the final nomogram model, we included variables with *P*-values < 0.05 from the previous multivariate logistic regression to minimize the risk of excluding potential confounders. The sample and limited number of variables included in the logistic regression-based nomogram prediction model ensured a low risk of overfitting. The scale of the line corresponding to each variable in the prediction model indicated the range of possible values for that variable, meanwhile, the length of the line indicated the effect of that factor on the outcome event. The points indicate the individual scores corresponding to each variable at different values to obtain the total score. A line was drawn downward based on the location of the total score and projected on the bottom scale, which determined the probability of fetal loss in patients with SLE with mild disease severity. The performance of the model was evaluated through internal validation and corrected for overfitting using bootstrap methods. Internal validation of the nomogram was performed using a bootstrap resampling method with 1000 iterations to assess predictive accuracy. The discriminative power of the predictive model was verified using the consistency index (C-index) and subject work characteristic curve (receiver operating characteristic [ROC] curve). The calibration curve of the model was evaluated by calculating the Brier score, with low Brier scores indicating improved model accuracy. Decision curve analysis (DCA) was performed to evaluate the clinical utility and net clinical benefit of the predictive model. Identification and calibration were assessed through bootstrapping with 1000 resamples.

### Statistical analysis

All tests were two-sided, and *P* < 0.05 was defined as statistically significant. For parameters with continuous data, normal distribution was expressed as the mean and standard deviation, and skewed distribution was expressed as the median and interquartile range (P25, and P75). Count data were expressed as ratios (%). Statistical computations were performed using SPSS software (version 22.0; SPSS, Chicago, IL, USA) and the R software package version 3.6.1 (https://www.r-project.org). Nomograms and calibration plots were generated using the rms package in the R software. Moreover, DCA was performed using the rmda package in the R software.

## Results

### APOs in patients with SLE with different disease activity

Records of 186 pregnancies from 175 patients with SLE collected at Shenzhen People's Hospital demonstrated that 149 of these pregnancies (80.1%) had mild disease severity before conception. Among patients with SLE in the mild disease severity cohort (*n* = 58), 38.9% experienced an APO compared to 56.8% in the moderate/severe disease severity cohort, and the difference between the two datasets was statistically significant (*P* < 0.05). The fetal loss occurred in 22.8% of pregnancy outcome records in one group and 24.3% in the other group; however, the difference was not statistically significant (*P* = 0.89). The APOs observed in patients with SLE with different disease severities before conception are described in detail in Table 2.

**Table 2:** Adverse pregnancy outcome of mild disease severity cohort and moderate/severe disease severity cohort

Category	Mild disease severity Cohort (*n* = 149)	Moderate/Severe disease severity Cohort (*n* = 37)	*P* value
Fetal loss	34(22.8%)	9(24.3%)	0.894
Spontaneous abortion	13(8.7%)	1(2.7%)	0.371
Stillbirth⃞	5(3.4%)	2(5.4%)	0.917
Therapeutic abortion	14(9.4%)	5(13.5%)	0.662
Early neonatal death	2(1.3%)	1(2.7%)	0.488
Other adverse pregnancy outcome			
Lupus flares	15(10.1%)	6( 13.5%)	0.443
Emergency cesarean section	5(3.4%)	3(8.1%)	0.411
Pre-eclampsia	6(4.0%)	4(10.8%)	0.021
Preterm birth (< 37 weeks)	4(2.7%)	2(5.4%)	0.750
Fetal growth restriction	7(4.7%)	2(5.4%)	0.677
All adverse pregnancy outcome	58(38.9%)	21(56.8%)	0.007

### Feature selection and traditional and dynamic nomogram model establishment

Table 3 demonstrates the outcomes of univariate and multivariate logistic regression analyses. Based on univariate analysis, SLE duration at conception, lupus nephritis (LN), history of therapeutic abortion, C3, lupus anticoagulant, IgG, serum ALB, CRP, PRO, HDL, and HCQ levels were significantly associated with the occurrence of fetal loss. The variance inflation factor was less than two, suggesting no significant multicollinearity among the four continuous variables (C3, IgG, ALB, and CRP) included in the multivariate logistic regression analysis (Supplementary Table 1). Multivariate analysis demonstrated that LN (odds ratio [OR] (95% confidence interval [CI]) 3.68 (1.14, 11.83), *P* = 0.029), C3 (OR (95% CI) 0.10 (0.01, 0.99), *P* = 0.049), IgG (OR (95% CI) 1.18 (1.03, 1.35), *P* = 0.015), serum ALB (OR (95% CI) 0.86 (0.79, 0.99), *P* = 0.029), CRP (OR (95% CI) 1.02 (1.01, 1.04), *P* = 0.012) and HCQ (OR (95% CI) 0.28 (0.09, 0.92), *P* = 0.036) were independent risk factors. A combination of these factors accurately predicted fetal loss (Table 3).

**Table 3:** Univariate and multivariate analysis of factors predicting fetal loss in the training cohort of SLE patients with low disease activity (*N* = 149)

Factors			Univariate analysis			Multivariate analysis		
			OR	95% CI	*P* value	OR	95% CI	*P* value
Baseline characteristics	Age(year)		1.74	0.99 ~ 1.16	0.083			
	Region	(Urban/Rural)	0.48	0.22 ~ 1.05	0.065			
	BMI before pregnant (kg/m2)		1.03	0.89 ~ 1.18	0.730			
Other chronic disease	Prepregnancy diabetes		3.50	0.47 ~ 25.84	0.219			
	Prepregnancy hypertension		1.53	0.17 ~ 13.54	0.703			
Previous SLE clinical features	Age of SLE diagnosis(years)		0.98	0.90 ~ 1.06	0.557			
	SLE duration at conception (years)		1.13	1.04 ~ 1.24	**0.007**	1.04	0.92 ~ 1.17	0.561
System manifestation	Arthritis	(Yes/No)	1.76	0.68 ~ 4.52	0.243			
	Cutaneous lesion	(Yes/No)	0.97	0.44 ~ 2.10	0.923			
	Hematological disorder	(Yes/No)	2.20	0.83 ~ 5.80	0.112			
	Serositis	(Yes/No)	5.42	0.87 ~ 33.88	0.071			
	Nephritis	(Yes/No)	4.74	2.05 ~ 10.99	** < 0.001**	3.68	1.14 ~ 11.83	**0.029**
	SLADAI before conception		1.15	0.95 ~ 1.40	0.144			
Obstetric history	Primigravida	(Yes/No)	0.88	0.37 ~ 2.09	0.739			
	History of therapeutic abortion	(Yes/No)	4.24	1.90 ~ 13.10	**0.001**	2.22	0.57 ~ 8.68	0.253
	History of recurrent miscarriages ≥ 2	(Yes/No)	3.20	0.87 ~ 15.53	0.078			
Laboratory data	ANA	(P/N)	1.88	0.40 ~ 8.86	0.423			
	Anti-Ro/SSA	(P/N)	2.04	0.94 ~ 4.43	0.073			
	Anti-La/ SSB	(P/N)	1.53	0.54 ~ 4.35	0.424			
	Anti-dsDNA	(P/N)	2.00	0.92 ~ 4.35	0.079			
	Anti-Sm	(P/N)	1.22	0.51 ~ 2.93	0.659			
	C3 (g/L)		0.10	0.01 ~ 0.31	**0.001**	0.10	0.01 ~ 0.99	**0.049**
	C4 (g/L)		1.66	0.66 ~ 4.16	0.279			
	Lupus anticoagulant	(P/N)	2.45	1.07 ~ 5.61	**0.034**	1.11	0.34 ~ 3.60	0.868
	aCL-IgG	(P/N)	0.55	0.06 ~ 4.70	0.581			
	aCL-IgM	(P/N)	0.45	0.10 ~ 2.07	0.303			
Hyperimmunoglobulin	IgA (g/L)		1.36	0.95 ~ 1.94	0.091			
	IgG (g/L)		1.10	1.01 ~ 1.21	**0.030**	1.18	1.03 ~ 1.35	**0.015**
	IgM(g/L)		1.99	0.99 ~ 3.92	0.053			
	HB (g/L)		1.01	0.99 ~ 1.03	0.529			
	WBC(× 109/L)		0.81	0.72 ~ 1.02	0.073			
	PLT(× 109/L)		1.00	1.00 ~ 1.03	0.745			
	Serum albumin(g/L)		0.88	0.83 ~ 0.96	**< 0.001**	0.86	0.79 ~ 1.00	**0.044**
	ESR (mm/h)		1.01	1.00 ~ 1.03	0.084			
	CRP(mg/L)		1.02	1.00 ~ 1.03	**0.042**	1.02	1.01 ~ 1.04	**0.012**
	24-h PRO(g/L)		1.15	0.90 ~ .1.46	0.259			
	PRO	(P/N)	2.64	1.21 ~ 5.76	**0.015**	1.96	0.60 ~ 6.38	0.263
	TC (mmol/L)		1.02	0.98 ~ 1.06	0.467			
	TG (mmol/L)		0.87	0.61 ~ 1.25	0.455			
	HDL(mmol/L)		0.31	0.11 ~ 0.88	**0.028**	0.77	0.16 ~ 3.74	0.748
	LDL(mmol/L)		1.23	0.90 ~ 1.68	0.196			
Drugs taken at the onset of pregnancy	Prednisone	(Yes/No)	1.62	0.09 ~ 3.80	0.267			
	Hydroxychloroquine;	(Yes/No)	2.50	1.15 ~ 5.83	**0.022**	0.28	0.09 ~ 0.92	**0.036**
	Immunosuppressants	(Yes/No)	0.53	0.19 ~ 1.50	0.231			
	Aspirin	(Yes/No)	1.13	0.29 ~ 4.43	0.862			

*CI* confidence interval, *OR* odd ratio, *P* Positive, *N* Negative

**Fig. 2:**
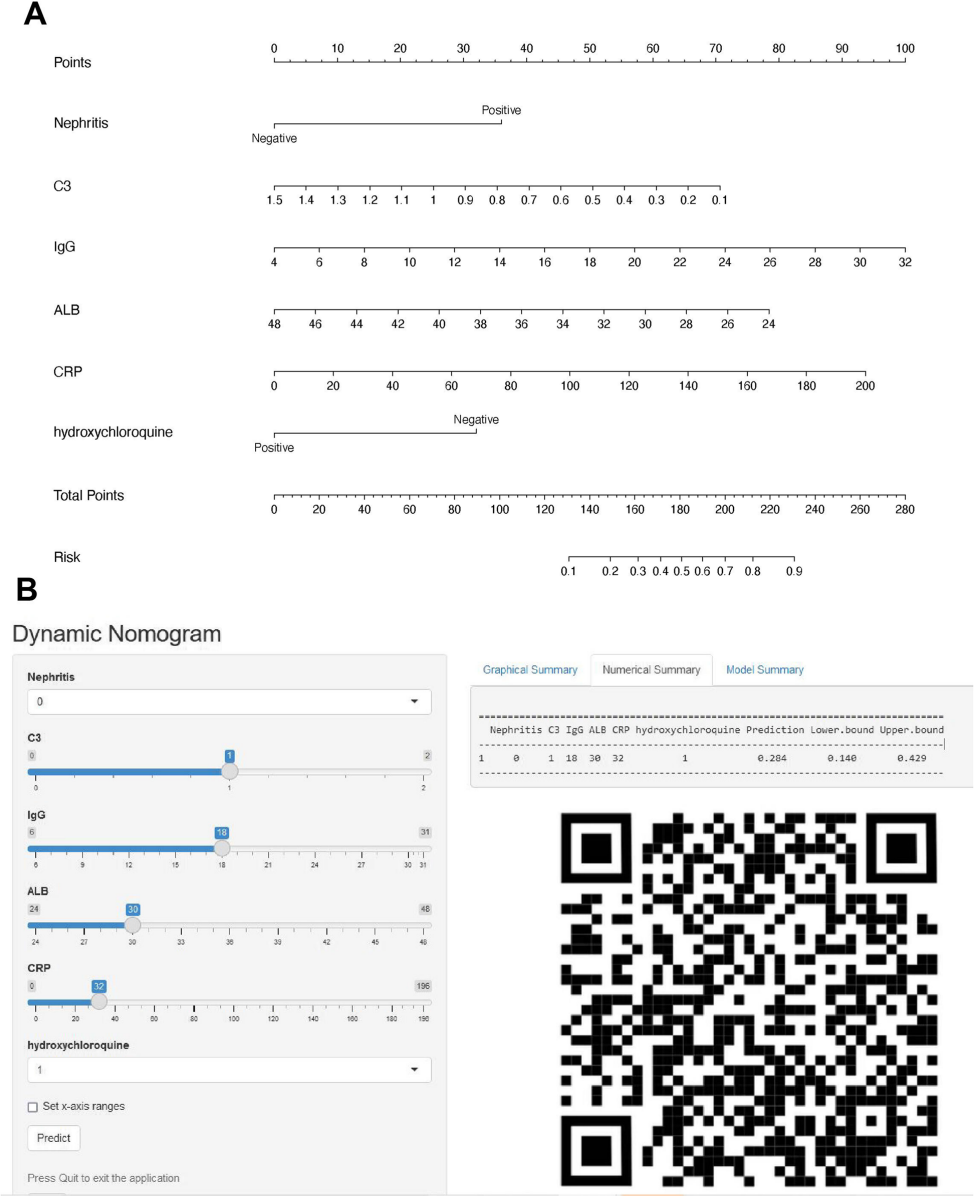
Nomogram for predictive model. **A** The nomogram of low disease activity SLE patients with 6 clinical factors predicting pregnancy outcome was established. **B** Scan this QR code to view the dynamic nomogram, or visit https://yanranchen.shinyapps.io/dynnomapp

We then conducted a multivariate logistic regression analysis of six independent factors to create a nomogram for predicting fetal loss outcomes in patients with SLE with mild disease severity (Fig. 2A). The (top) points were obtained based on predictor contributions, whereas the (bottom) points were normalized to the probability of fetal loss. The prediction points were located on the highest-point scale corresponding to patient variables. The points corresponding to each variable were summed, and the predicted values were obtained at the bottom of the nomogram. The total number on the bottom scale represents the predicted probability of fetal loss. The dynamic nomogram is available online at https://yanranchen.shinyapps.io/dynnomapp. After entering the values of the six variables on the web page, we obtained the exact probability of fetal loss for the patient, which was 28.4% (Fig. 2B).

If the dynamic nomogram modeling tool was not accessible, the prediction results were manually calculated based on the nomogram plot (Fig. 2A). For instance, a patient with no history of LN before conception, taking HCQ, had a C3 level of 1 g/L, an IgG level of 18 g/L, a serum ALB level of 30 g/L, and a CRP level of 32 mg/L. The scores for each predictor were 0, 0, 25, 50, 60, and 14, respectively. A total score of 149 was obtained, corresponding to a probability of 25%. Therefore, the predicted probability of fetal loss outcome in this patient was approximately 25%.

### Calibration and validation of the nomogram

**Fig. 3:**
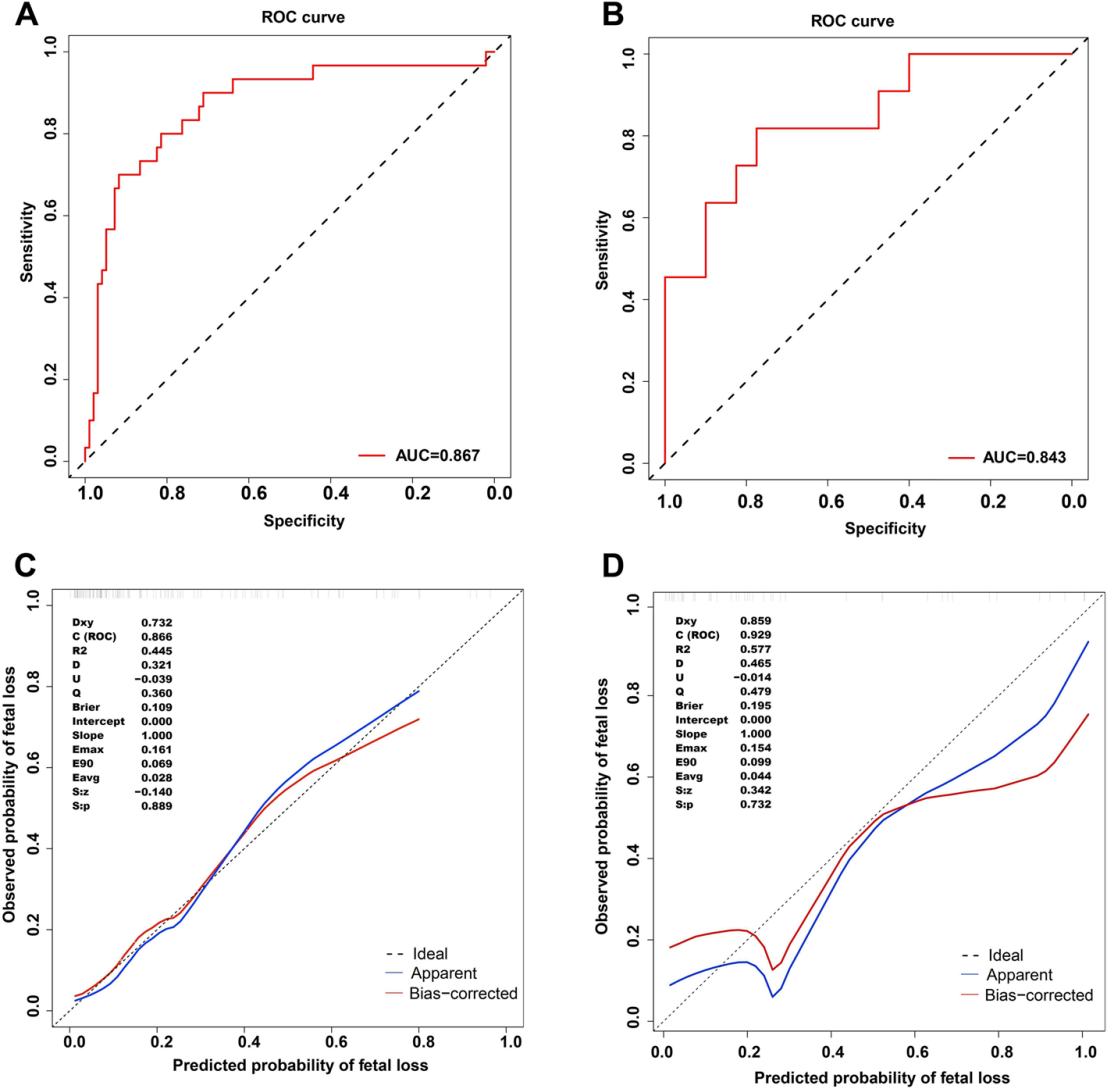
ROC curve analyses and Calibration curves analysis of the nomogram model. AUC: area under the curve;ROC: receiver operating characteristic. **A** ROC curves of the nomogram model in the traincohort cohort. **B** ROC curves of the nomogram model in the validation cohort. **C** Calibration curve of the nomogram model in training cohort. **D** Calibration curve of the nomogram model in validation cohort.The y-axis represents the observed probability of fetal loss. The x-axis represents the Predicted probability of fetal loss. The diagonal dotted line represents the ideal model with the best prediction. The solid line represents the performance of the nomograms, the proximity of which to the diagonal dotted line represents the prediction abilities of the two models

**Fig. 4:**
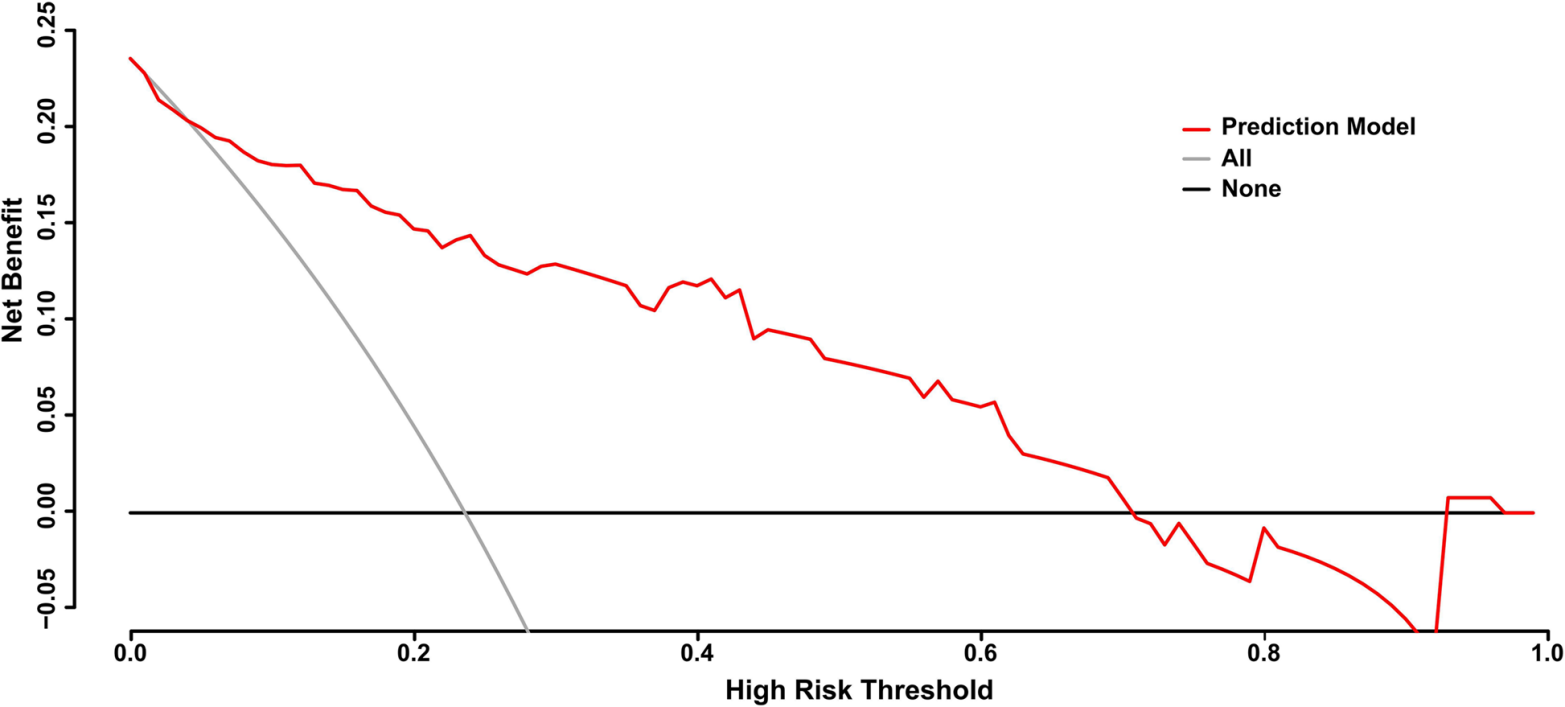
Decision curve analysis for the nomogram model. The y-axis measures the net benefit. The red line represents the predict nomogram. The black solid line represents the assumption that no patients have fetal loss and the thin grey solid line indicates the hypothesis that all patients have fetal loss. The decision curves indicate that if the threshold probability is 10–70%, the nomogram model constructed for prediction of fetal loss in SLE patients was benefit

ROC analysis demonstrated considerably good predictive performance of the nomogram in the training cohort, with an area under the ROC curve (AUC) of 0.867 (95% CI: 0.787–0.947) (Fig. 3A). The calibration curves exhibited good agreement in predicting fetal loss outcomes, closely resembling the ideal calibration model. Therefore, indicating good calibration (Fig. 3C). The low Brier score (0.109) confirmed the excellent predictive ability of the nomogram. In the validation cohort, the value of AUC (0.843,95% CI: 0.711–0.975) (Fig. 3B) and the calibration curve (Fig. 3D) indicated good discriminative ability of the model. The overall predictions, as measured by Brier scores (0.195), were also good for the validation model. The DCA of the nomogram model revealed a substantial net benefit across the predicted probability range of 10% to 70% (Fig. 4).

## Discussion

Studies consistently indicate that fetal loss rates are high in pregnant women with increased disease activity before conception and in those with SLE complicated by APS [18, 19]. However, does mild disease severity in pregnant women with SLE traditionally prevent the outcome of fetal loss? Our study, which reviewed the pregnancy experiences of patients with SLE, demonstrated results that were different from those of the clinical experience. Patients with SLE who meet the criteria for mild disease severity as defined by the 2019 update of EULAR management recommendations still face an elevated risk of fetal loss. In our study, patients with mild disease severity were less likely to have an APO than those with moderate or severe disease severity (38.9% vs. 56.8%, respectively), with the probability of fetal loss being 22.8% vs. 24.3%. Disease severity in patients before conception does not seem to provide a predictive signal for fetal loss.

This study identified that 22.8% of patients with mild disease severity and not classified as APS reported higher fetal loss than that documented in previous studies. Our study aimed to develop a reliable nomogram model for predicting the probability of fetal loss by analyzing the characteristics of 149 women with mild disease severity before conception at our institution. Previous reports have described several potential risk factors for APOs in patients with SLE [20, 21]. Our study demonstrated the SLE duration at conception, LN, history of therapeutic abortion, C3, lupus anticoagulant, IgG, serum ALB, CRP, PRO, HDL, and HCQ were identified as potential correlates of fetal loss in pregnant women with SLE with mild disease severity. Among these, we identified the most suitable combination of the following factors: LN, C3, IgG, ALB, CRP, and HCQ. The combination of these factors accurately predicted fetal loss. Our results demonstrated that this model has a high predictive accuracy. In addition, validation confirmed that the model had good discrimination and calibration abilities.

To our knowledge, no other studies have targeted predictive models for fetal loss outcomes in pregnant women with SLE and mild disease severity. Most existing studies have focused on analyzing individual factors affecting APOs in women with SLE. To date, serologic and clinical outcomes predicting fetal loss have been controversial, and studies have concluded that the risk of fetal loss is significantly high if APL is detected in the maternal circulation [22]. Gabriella Moroni et al., in a prospective study of pregnancy outcomes in patients with LN, demonstrated that fetal loss occurred in 38% of lupus anticoagulant-positive pregnancies compared to that in 1.7% of lupus anticoagulant-negative pregnancies [23]. Between 2003 and 2011, Michael D. et al. demonstrated that APO occurred at a lower rate in lupus anticoagulant-negative women than in those who demonstrated lupus anticoagulant-positivity (8% vs. 43%, *P* = 0.02). Furthermore, aCL, IgM and anti-β2GPI antibodies did not predict the occurrence of APO [24]. The study recognized lupus anticoagulants as a major predictor of APO after 12 weeks of gestation. This is consistent with our finding that lupus anticoagulant use is a risk factor for fetal loss in pregnant women with SLE who demonstrate low disease activity before conception. In 2018, Stephen et al. conducted the first longitudinal study on HCQ drug concentrations during pregnancy [25]. The results demonstrated a significantly higher incidence of preterm delivery and low gestational age in patients with SLE with low serum HCQ levels (≤ 100 ng/mL) than in those with HCQ levels > 100 ng/mL. Our study demonstrates that maternal use of HCQ before conception helps reduce the possibility of fetal loss, which may be related to the fact that HCQ helps reduce the incidence of lupus flares during pregnancy [26, 27]. The Predictors of Pregnancy Outcomes: Biomarkers in Antiphospholipid Antibody Syndrome and Systemic Lupus Erythematosus (PROMISSE) study is the largest multicenter, multi-racial, and multi-ethnic prospective clinical evaluation study of laboratory predictors of APL and/or SLE inactivity or mild APO in women [28]. Mimi Y et al. utilized data from the PROMISSE study to investigate whether complement activation predicted APOs in patients with SLE and/or antiphospholipid antibodies [29]. Previous studies have reported varying findings regarding the impact of outcomes of C3 levels during pregnancy in patients with SLE. In a study of complement levels in 530 women with SLE between 1992 and 2003, Ramos-Casals et al. discovered that patients with low complement levels had similar rates of pregnancy miscarriages as the rates observed in those with normal complement levels [30]. Conversely, the study published by Cortes et al. in 2002 demonstrated a significant correlation between low complement levels detected at the initial visit or every 3 months and the outcome of miscarriage and stillbirth [31]. In our study, complement levels during pregnancy were independent risk factors for predicting fetal loss. Additionally, LN is strongly correlated with the risk of fetal loss in pregnant women with SLE [28, 32, 33]. Gabriella Moroni et al. reviewed 37 studies, including 2751 pregnancy outcomes in 1842 women with LN. They reported a treatment-related miscarriages occurred in 5.9% of cases, spontaneous abortion in 16%, stillbirth in 3.6%, and neonatal death in 2.5% [23]. Rates of fetal loss in available studies on mothers with LN lupus nephritis range from 13 to 35%, with worse pregnancy outcomes in African American and Hispanic women, with fetal loss rates of 27.4% and 20.6%, respectively, compared with 5% in Caucasian patients [23, 34–36]. Unfortunately, comprehensive studies documenting pregnancy outcomes in Chinese women with LN are lacking. One of the most common complications of SLE, LN was a risk factor for fetal loss in our study. Several studies have used red blood cell distribution width to assess renal function and the extent of renal injury in autoimmune diseases. In contrast, the mechanism by which eclampsia occurs in LN remains unclear, and reliable biomarkers need to be identified. Studies have demonstrated that anti-LAMP-2 antibodies are useful for the differential diagnosis of vascular injury in autoimmune diseases [37, 38].

The most recent study on miscarriage prediction models for patients with SLE in China was conducted by Wu et al. [39] who utilized data from 338 patients with SLE between 2011 and 2017 to construct a pregnancy loss prediction model. The SLEDAI-2K was utilized [40] as a valid measure of disease activity. Patients were divided into planned and unplanned pregnancy groups, with the planned pregnancy group including patients with SLE in control or remission for more than 6 months before conception, and the unplanned pregnancy group including patients with active lupus before conception or new-onset SLE during pregnancy. This does not align with our classification criteria. Of note, 24-h PRO quantification, complement C3 level, and unplanned pregnancy were considered independent predictors. The model was constructed using stepwise regression analysis with an AUC value of 0.829 (95% confidence interval [CI], 0.744–0.91). Our study identified that 22.8% of patients with mild disease severity and those not classified as APS experienced fetal loss. Therefore, identifying patients with low-risk SLE during pregnancy for early management is necessary. This study integrated multiple predictors using a nomogram prediction model to achieve personalized and accurate prediction of the probability of fetal loss. In contrast, we focused on women with SLE who had mild disease severity before conception, examined multiple factors potentially associated with straight fetal loss, and created column line plots by screening for the most appropriate combination of factors. The nomogram is a practical visualization tool that facilitates the identification of patients at high risk of fetal loss and quantifies individual risk. The proposed model aims to empower clinicians to personalize and accurately assess the early risk of fetal loss, thereby mitigating pregnancy risks for women with SLE. In practice, focusing not only on patients at high risk of traditional fetal loss, such as those with advanced pre-pregnancy disease activity, LN, and APS but also on the risk of pregnancy loss in patients with SLE at low prenatal risk is essential.

Our study had several limitations owing to the small number of pregnant women with SLE. For instance, this was a two-center retrospective study conducted in Shenzhen. Therefore, we sought external validation in additional central studies. Moreover, this was a retrospective study and the electronic medical records lacked details. In contrast, detailed medication records related to SLE and records of patients' previous pregnancy outcomes may provide detailed information for predicting fetal loss, which is beyond the scope of this study [41]. Finally, our study included only Chinese patients with SLE, and the results may not be generalizable to other populations. As mentioned previously, predicting the risk of fetal loss in patients with SLE with mild disease severity remains challenging due to the lack of validated predictive models. Further validation using data from other countries would help to improve the generalizability of the model and expand the population to which the nomogram model applies. Although acknowledging the population-specific limitations of the predictive model developed in this study, its broader application could allow for individualized management of pregnant women with SLE. This approach considers the relative risks and benefits to both the patient and fetus, empowering physicians to make more informed, data-driven decisions in patient management.

## Conclusions

In conclusion, this dynamic nomogram model offers a straightforward, illustrative, clinically friendly, and useful predictive tool that may be used to address the most critical concerns for women carrying low-risk SLE pregnancies. These findings may offer strategies to reduce the risk of fetal loss in patients with SLE.

### Supplementary Information

Supplementary Material 1.

## Data Availability

The datasets generated and/or analysed during the current study are not publicly available due individual privacy but are available from the corresponding author on reasonable request.

## Abbreviations

SLE: Systemic lupus erythematosus
APS: Anti-phospholipid syndrome
ACR: American College of Rheumatology
SLICC: Systemic Lupus International Collaboration Clinic Classification
BMI: Body mass index
HB: Hemoglobin
WBC: White blood cells
PLT: Platelets
CRP: C-reactive protein
PRO: Urine protein
24-h PRO: 24-Hour urine protein
ESR: Hematocrit
ALB: Albumin
TC: Cholesterol
TG: Triglycerides
LDL: Low-density lipoprotein
HDL: High-density lipoprotein
IgA: Immunoglobulin A
IgM: Immunoglobulin M
IgG: Immunoglobulin G
C3: Complement 3
C4: Complement 4
ANA: Antinuclear antibodies
aCL: Anticardiolipin
CsA: Cyclosporine A
TAC: Tacrolimus
MMF: Mofetil
AZA: Azathioprine
MTX: Methotrexate
SLEDAI: SLE Disease Activity Index
LA: Lupus anticoagulant
APO: Adverse pregnancy outcome
C-index: Consistency index
ROC: Receiver operating characteristic
AUC: Area under the ROC curve
DCA: Decision curve analysis
IQR: Interquartile range

## References

[1] Carter EE, Barr SG, Clarke AE. The global burden of SLE: prevalence, health disparities and socioeconomic impact. Nat Rev Rheumatol. 2016;12(10):605–20. 27558659 10.1038/nrrheum.2016.137

[2] Peart E, Clowse ME. Systemic lupus erythematosus and pregnancy outcomes: an update and review of the literature. Curr Opin Rheumatol. 2014;26(2):118–23. 24419751 10.1097/BOR.0000000000000030

[3] Medina-Quinones CV, Ramos-Merino L, Ruiz-Sada P, Isenberg D. Analysis of complete remission in systemic lupus erythematosus patients over a 32-year period. Arthritis Care Res (Hoboken). 2016;68(7):981–7. 26554745 10.1002/acr.22774

[4] Oktem O, Yagmur H, Bengisu H, Urman B. Reproductive aspects of systemic lupus erythematosus. J Reprod Immunol. 2016;117:57–65. 27474801 10.1016/j.jri.2016.07.001

[5] Oktem O, Guzel Y, Aksoy S, Aydin E, Urman B. Ovarian function and reproductive outcomes of female patients with systemic lupus erythematosus and the strategies to preserve their fertility. Obstet Gynecol Surv. 2015;70(3):196–210. 25769434 10.1097/OGX.0000000000000160

[6] Vinet E, Labrecque J, Pineau CA, Clarke AE, St-Pierre Y, Platt R, Bernatsky S. A population-based assessment of live births in women with systemic lupus erythematosus. Ann Rheum Dis. 2012;71(4):557–9. 22084391 10.1136/annrheumdis-2011-200276

[7] Mehta B, Luo Y, Xu J, Sammaritano L, Salmon J, Lockshin M, Goodman S, Ibrahim S. Trends in maternal and fetal outcomes among pregnant women with systemic lupus erythematosus in the United States: a cross-sectional analysis. Ann Intern Med. 2019;171(3):164–71. 31284305 10.7326/M19-0120

[8] Buyon JP, Kim MY, Guerra MM, Lu S, Reeves E, Petri M, Laskin CA, Lockshin MD, Sammaritano LR, Branch DW, et al. Kidney outcomes and risk factors for nephritis (Flare/De Novo) in a multiethnic cohort of pregnant patients with lupus. Clin J Am Soc Nephrol. 2017;12(6):940–6. 28400421 10.2215/CJN.11431116 PMC5460714

[9] Li X, Shopit A, Wang J. Biochemical and clinical predictors in pregnant women with antiphospholipid syndrome and systemic lupus erythematosus: comprehensive update. Arch Gynecol Obstet. 2021;304(5):1153–60. 34390384 10.1007/s00404-021-06178-5

[10] Andreoli L, Bertsias GK, Agmon-Levin N, Brown S, Cervera R, Costedoat-Chalumeau N, Doria A, Fischer-Betz R, Forger F, Moraes-Fontes MF, et al. EULAR recommendations for women’s health and the management of family planning, assisted reproduction, pregnancy and menopause in patients with systemic lupus erythematosus and/or antiphospholipid syndrome. Ann Rheum Dis. 2017;76(3):476–85. 27457513 10.1136/annrheumdis-2016-209770 PMC5446003

[11] Kim JW, Jung JY, Kim HA, Yang JI, Kwak DW, Suh CH. lupus low disease activity state achievement is important for reducing adverse outcomes in pregnant patients with systemic lupus erythematosus. J Rheumatol. 2021;48(5):707–16. 33060317 10.3899/jrheum.200802

[12] Kwok LW, Tam LS, Zhu T, Leung YY, Li E. Predictors of maternal and fetal outcomes in pregnancies of patients with systemic lupus erythematosus. Lupus. 2011;20(8):829–36. 21543513 10.1177/0961203310397967

[13] Tektonidou MG, Andreoli L, Limper M, Amoura Z, Cervera R, Costedoat-Chalumeau N, Cuadrado MJ, Dorner T, Ferrer-Oliveras R, Hambly K, et al. EULAR recommendations for the management of antiphospholipid syndrome in adults. Ann Rheum Dis. 2019;78(10):1296–304. 31092409 10.1136/annrheumdis-2019-215213 PMC11034817

[14] Lazzaroni MG, Dall’Ara F, Fredi M, Nalli C, Reggia R, Lojacono A, Ramazzotto F, Zatti S, Andreoli L, Tincani A. A comprehensive review of the clinical approach to pregnancy and systemic lupus erythematosus. J Autoimmun. 2016;74:106–17. 27377453 10.1016/j.jaut.2016.06.016

[15] He X, Jiang D, Wang Z, Li Y, Wang J, Xu D, Chen J, Liu X, Gao G. Clinical features of new-onset systemic lupus erythematosus during pregnancy in Central China: a retrospective study of 68 pregnancies. Clin Rheumatol. 2021;40(6):2121–31. 33064224 10.1007/s10067-020-05452-2

[16] Erkan D, Sammaritano L. New insights into pregnancy-related complications in systemic lupus erythematosus. Curr Rheumatol Rep. 2003;5(5):357–63. 12967517 10.1007/s11926-003-0021-9

[17] Fanouriakis A, Kostopoulou M, Alunno A, Aringer M, Bajema I, Boletis JN, Cervera R, Doria A, Gordon C, Govoni M, et al. 2019 update of the EULAR recommendations for the management of systemic lupus erythematosus. Ann Rheum Dis. 2019;78(6):736–45. 30926722 10.1136/annrheumdis-2019-215089

[18] Sammaritano LR. Management of systemic lupus erythematosus during pregnancy. Annu Rev Med. 2017;68:271–85. 27686021 10.1146/annurev-med-042915-102658

[19] Sammaritano LR, Bermas BL, Chakravarty EE, Chambers C, Clowse MEB, Lockshin MD, Marder W, Guyatt G, Branch DW, Buyon J, et al. 2020 American college of rheumatology guideline for the management of reproductive health in rheumatic and musculoskeletal diseases. Arthritis Rheumatol. 2020;72(4):529–56. 32090480 10.1002/art.41191

[20] Tan Y, Yang S, Liu Q, Li Z, Mu R, Qiao J, Cui L. Pregnancy-related complications in systemic lupus erythematosus. J Autoimmun. 2022;132:102864. 35872104 10.1016/j.jaut.2022.102864

[21] Society for Maternal-Fetal Medicine, Electronic address pso, Silver R, Craigo S, Porter F, Osmundson SS, Kuller JA, Norton ME. Society for Maternal-Fetal Medicine Consult Series #64: Systemic lupus erythematosus in pregnancy. Am J Obstet Gynecol. 2023;228(3):B41–60. 36084704 10.1016/j.ajog.2022.09.001

[22] Hong J, Zhu B, Cai X, Liu S, Liu S, Zhu Q, Aierken X, Aihemaiti A, Wu T, Li N. Assessment of the association between red blood cell distribution width and disease activity in patients with systemic vasculitis. Exp Ther Med. 2021;22(1):691. 33986856 10.3892/etm.2021.10123 PMC8112135

[23] Moroni G, Doria A, Giglio E, Tani C, Zen M, Strigini F, Zaina B, Tincani A, de Liso F, Matinato C, et al. Fetal outcome and recommendations of pregnancies in lupus nephritis in the 21st century. A prospective multicenter study. J Autoimmun. 2016;74:6–12. 27496151 10.1016/j.jaut.2016.07.010

[24] Lockshin MD, Kim M, Laskin CA, Guerra M, Branch DW, Merrill J, Petri M, Porter TF, Sammaritano L, Stephenson MD, et al. Prediction of adverse pregnancy outcome by the presence of lupus anticoagulant, but not anticardiolipin antibody, in patients with antiphospholipid antibodies. Arthritis Rheum. 2012;64(7):2311–8. 22275304 10.1002/art.34402 PMC3357451

[25] Balevic SJ, Cohen-Wolkowiez M, Eudy AM, Green TP, Schanberg LE, Clowse MEB. Hydroxychloroquine levels throughout pregnancies complicated by rheumatic disease: implications for maternal and neonatal outcomes. J Rheumatol. 2019;46(1):57–63. 30275257 10.3899/jrheum.180158 PMC6314916

[26] Clowse ME, Magder L, Witter F, Petri M. Hydroxychloroquine in lupus pregnancy. Arthritis Rheum. 2006;54(11):3640–7. 17075810 10.1002/art.22159

[27] Ruiz-Irastorza G, Ramos-Casals M, Brito-Zeron P, Khamashta MA. Clinical efficacy and side effects of antimalarials in systemic lupus erythematosus: a systematic review. Ann Rheum Dis. 2010;69(1):20–8. 19103632 10.1136/ard.2008.101766

[28] Buyon JP, Kim MY, Guerra MM, Laskin CA, Petri M, Lockshin MD, Sammaritano L, Branch DW, Porter TF, Sawitzke A, et al. Predictors of pregnancy outcomes in patients with lupus: a cohort study. Ann Intern Med. 2015;163(3):153–63. 26098843 10.7326/M14-2235 PMC5113288

[29] Kim MY, Guerra MM, Kaplowitz E, Laskin CA, Petri M, Branch DW, Lockshin MD, Sammaritano LR, Merrill JT, Porter TF, et al. Complement activation predicts adverse pregnancy outcome in patients with systemic lupus erythematosus and/or antiphospholipid antibodies. Ann Rheum Dis. 2018;77(4):549–55. 29371202 10.1136/annrheumdis-2017-212224 PMC6037302

[30] Ramos-Casals M, Campoamor MT, Chamorro A, Salvador G, Segura S, Botero JC, Yague J, Cervera R, Ingelmo M, Font J. Hypocomplementemia in systemic lupus erythematosus and primary antiphospholipid syndrome: prevalence and clinical significance in 667 patients. Lupus. 2004;13(10):777–83. 15540510 10.1191/0961203304lu1080oa

[31] Cortes-Hernandez J, Ordi-Ros J, Paredes F, Casellas M, Castillo F, Vilardell-Tarres M. Clinical predictors of fetal and maternal outcome in systemic lupus erythematosus: a prospective study of 103 pregnancies. Rheumatology (Oxford). 2002;41(6):643–50. 12048290 10.1093/rheumatology/41.6.643

[32] Fanouriakis A, Kostopoulou M, Cheema K, Anders HJ, Aringer M, Bajema I, Boletis J, Frangou E, Houssiau FA, Hollis J, et al. 2019 Update of the Joint European League Against Rheumatism and European Renal Association-European Dialysis and Transplant Association (EULAR/ERA-EDTA) recommendations for the management of lupus nephritis. Ann Rheum Dis. 2020;79(6):713–23. 32220834 10.1136/annrheumdis-2020-216924

[33] Schreiber K, Stach K, Sciascia S. Lupus nephritis and pregnancy outcome. Autoimmun Rev. 2017;16(4):433–4. 28167150 10.1016/j.autrev.2017.02.001

[34] Palma Dos Reis CR, Cardoso G, Carvalho C, Nogueira I, Borges A, Serrano F. Prediction of Adverse Pregnancy Outcomes in Women with Systemic Lupus Erythematosus. Clin Rev Allergy Immunol. 2020;59(3):287–94. 31444781 10.1007/s12016-019-08762-9

[35] Moroni G, Doria A, Giglio E, Imbasciati E, Tani C, Zen M, Strigini F, Zaina B, Tincani A, Gatto M, et al. Maternal outcome in pregnant women with lupus nephritis. A prospective multicenter study. J Autoimmun. 2016;74:194–200. 27373903 10.1016/j.jaut.2016.06.012

[36] Braga A, Barros T, Faria R, Marinho A, Rocha G, Farinha F, Neves E, Vasconcelos C, Braga J. Systemic lupus erythematosus and pregnancy: a Portuguese case-control study. Clin Rev Allergy Immunol. 2022;62(2):324–32. 34519994 10.1007/s12016-021-08893-y

[37] Cai X, Zhu Q, Wu T, Zhu B, Liu S, Liu S, Aierken X, Ahmat A, Li N. Association of circulating resistin and adiponectin levels with Kawasaki disease: a meta-analysis. Exp Ther Med. 2020;19(2):1033–41. 32010266 10.3892/etm.2019.8306 PMC6966156

[38] Zhu B, Cai X, Zhu Q, Wu T, Liu S, Liu S, Hong J, Li N. The association of serum anti-lysosomal-associated membrane protein-2 antibody with vasculitis combined with hypertension. Int J Hypertens. 2022;2022:9656560. 35356030 10.1155/2022/9656560 PMC8960034

[39] Wu J, Zhang WH, Ma J, Bao C, Liu J, Di W. Prediction of fetal loss in Chinese pregnant patients with systemic lupus erythematosus: a retrospective cohort study. BMJ Open. 2019;9(2):e023849. 30755448 10.1136/bmjopen-2018-023849 PMC6377554

[40] Gladman DD, Ibanez D, Urowitz MB. Systemic lupus erythematosus disease activity index 2000. J Rheumatol. 2002;29(2):288–91. 11838846

[41] Nahal SK, Selmi C, Gershwin ME. Safety issues and recommendations for successful pregnancy outcome in systemic lupus erythematosus. J Autoimmun. 2018;93:16–23. 30056945 10.1016/j.jaut.2018.07.016

