# Supplementary material for: Construction and application of fetal loss risk model in systemic lupus erythematosus patients with mild disease severity
Source: BMC Pregnancy Childbirth. 2024 Jul 23;24:497. doi: 10.1186/s12884-024-06679-6 (PMC11264457; doi:10.1186/s12884-024-06679-6)
Supplement: Supplementary file 1 — Supplementary Material 1. [file 12884_2024_6679_MOESM1_ESM.docx]

| Factors | U Std. Coefficients | | Std. Coefficients | Statistics | | R^2^ | DurbinWatson |
| --- | --- | --- | --- | --- | --- | --- | --- |
|  | *B* | *S.E* | *β* | Tol | VIF |  |  |
| C3 | -0.280 | 0.132 | -0.170 | 0.913 | 1.095 | 0.300 | 0.722 |
| IgG | 0.021 | 0.008 | 0.209 | 0.945 | 1.058 |  |  |
| ALB | -0.020 | 0.007 | -0.230 | 0.917 | 1.091 |  |  |
| CRP | 0.003 | 0.001 | 0.203 | 0.987 | 1.013 |  |  |

Supplement table1. The result of multicollinearity analysis.

U Std. Coefficients: Unstandardized Coefficients; Std. Coefficients: Standardized Coefficients; Tol: tolerance; VIF: variance inflation factor; Statistical methods: Multicollinearity analysis.
